# V2 Protein Enhances the Replication of Genomic DNA of Mulberry Crinkle Leaf Virus

**DOI:** 10.3390/ijms251910521

**Published:** 2024-09-29

**Authors:** Zhen-Ni Yin, Pei-Yu Han, Tao-Tao Han, Ying Huang, Jing-Jing Yang, Meng-Si Zhang, Miao Fang, Kui Zhong, Jian Zhang, Quan-You Lu

**Affiliations:** 1 School of Biotechnology, Jiangsu University of Science and Technology, Zhenjiang 212100, China; 221211801111@stu.just.edu.cn (Z.-N.Y.); 221211802107@stu.just.edu.cn (P.-Y.H.); iamtaoth@163.com (T.-T.H.); 231211801105@stu.just.edu.cn (Y.H.); 231111802115@stu.just.edu.cn (J.-J.Y.); 231211802117@stu.just.edu.cn (M.-S.Z.); fmmy0506@just.edu.cn (M.F.); kuizhong2008@163.com (K.Z.); 2 Key Laboratory of Silkworm and Mulberry Genetic Improvement, Ministry of Agriculture and Rural Affairs, Sericultural Scientific Research Center, Chinese Academy of Agricultural Sciences, Zhenjiang 212100, China

**Keywords:** geminivirus, mulberry crinkle leaf virus, V2, replication enhancer

## Abstract

Mulberry crinkle leaf virus (MCLV), identified in mulberry plants (*Morus alba* L.), is a member of the genus *Mulcrilevirus* in the family *Geminiviridae*. The functions of the V2 protein encoded by MCLV remain unclear. Here, *Agrobacterium*-mediated infectious clones of a wild-type MCLV vII (MCLV^WT^) and two V2 mutant MCLV vIIs, including MCLV^mV2^ (with a mutation of the start codon of the V2 ORF) and MCLV^dV2^ (5′-end partial deletion of the V2 ORF sequence), were constructed to investigate the roles of V2 both in planta and at the cellular level. Although all three constructs (pCA-1.1MCLV^WT^, pCA-MCLV^mV2^, and pCA-MCLV^dV2^) were able to infect both natural host mulberry plants and experimental tomato plants systematically, the replication of the MCLV^mV2^ and MCLV^dV2^ genomes in these hosts was significantly reduced compared to that of MCLV^WT^. Similarly, the accumulation of MCLV^mV2^ and MCLV^dV2^ in protoplasts of *Nicotiana benthamiana* plants was significantly lower than that of MCLV^WT^ either 24 h or 48 h post-transfection. A complementation experiment further confirmed that the decreased accumulation of MCLV in the protoplasts was due to the absence of V2 expression. These results revealed that MCLV-encoded V2 greatly enhances the level of MCLV DNA accumulation and is designated the replication enhancer protein of MCLV.

## 1. Introduction

*Geminiviridae* is a family of nonenveloped plant viruses characterized by twin icosahedral particles c. 18 × 30 nm in size. Their genomes consist of mono- or bipartite single-stranded, circular DNA (ssDNA) the sizes of 2.5–5.2 kb [1]. Currently, over 520 reported species of geminiviruses are classified into 14 genera of the family based on their genome structure, host range, insect vectors, and genetic evolutionary relationships. These 14 genera are the genus *Becurtovirus*, *Begomovirus*, *Capulavirus*, *Curtovirus*, *Eragrovirus*, *Grablovirus*, *Mastrevirus*, *Topocuvirus*, *Turncurtovirus*, *Citlodavirus*, *Maldovirus*, *Mulcrilevirus*, *Opunvirus*, and *Topilevirus* [1,2]. Geminiviruses have a wide host range and infect both dicotyledonous and monocotyledonous herbaceous plants, including important crops such as wheat [3], tomato [4], cassava, and maize [5], as well as economically important woody plants, such as grapevine [6,7,8], citrus [9], apple [10], mulberry [11], and paper mulberry [12], causing significant damage to global agricultural production and economic losses in various regions and countries [13]. The most effective management of diseases caused by geminiviruses is an integrated pest management approach that involves planting resistant cultivars, propagating virus-free plants, rogueing infected plants, and killing insect vectors [14].

Geminivirial genomes encode four to eight canonical proteins in both virion- and complementary-sense strands [1]. All geminiviruses encode a coat protein (CP) and are uniformly designated V1 for monopartite and AV1 for bipartite, one to two movement proteins (MPs), and a replication-associated protein (Rep). These proteins are involved in viral DNA replication, transcription, movement, and encapsulation in host plants [15,16,17]. Geminiviruses also encode genus-specific proteins. The functions of some genus-specific proteins have been confirmed experimentally. For example, C3, encoded by the geminiviruses of the genus *Begomovirus*, *Curtovirus*, *Maldovirus*, *Opunvirus*, *Topocuvirus*, and *Turncurtovirus,* is a replication enhancer (REn) protein that can increase the accumulation of genome DNA replication during the infection cycle [18,19]. V4, encoded by mulberry crinkle leaf virus (MCLV) in the genus *Mulcrilevirus,* can increase viral replication, and V5 is needed for MCLV infection in *Nicotiana benthamiana* [20]. C4, which is encoded by geminiviruses in the genus *Begomovirus*, *Opunvirus*, *Topocuvirus*, and *Turncurtovirus,* is a multifunctional protein that plays a role in suppressing the host’s gene silencing, hypersensitive response (HR), and salicylic acid (SA)-mediated defense, as well as conferring drought stress tolerance independent of abscisic acid in plants [21,22,23,24]. V3, an additional small protein encoded by tomato yellow leaf curl virus (TYLCV), can suppress the RNA silencing mechanism of the host [25,26]. In spite of the great progress in understanding the functions of geminivirus-encoded proteins, there are still some proteins, such as C3, which is encoded by geminiviruses in the genera *Capulavirus*, *Grablovirus*, and *Topilevirus*; maldovirus-encoded C4; and capulavirus-encoded V4:V2, which functions remain to be elucidated.

**Figure 1:**
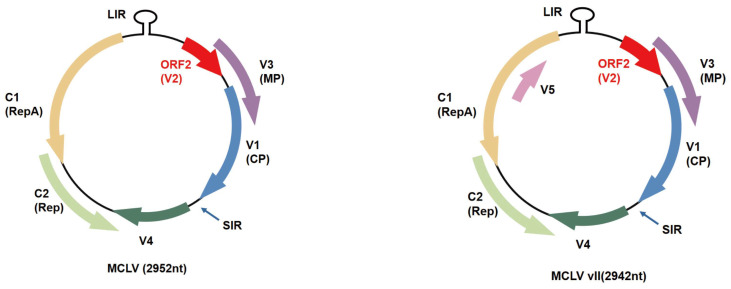
Genomic organization of the two MCLV variants.

MCLV, a representative species of the genus *Mulcrilevirus* (family *Geminiviridae*) [2,11], is transmitted naturally by a type of leafhopper, *Tautoneura mori***,** which occurs in mulberry fields or by grafting with MCLV-infected vegetative propagation materials [27,28] and infects natural mulberry (*Morus alba* L.) plants but does not induce visible symptoms on mulberry leaves [28,29]. The naturally occurring MCLV has two variants, MCLV and MCLV vII, and the greatest difference between these two variants is that MCLV vII encodes an additional V5 protein compared to MCLV (Figure 1) [27]. The monopartite genome of MCLV varies from 2920 nt to 2966 nt in size, depending on different haplotypes, containing a large intergenic region (LIR), a polyadenylation signal of approximately 37 nucleotides between the V1 and V4 open reading frames (ORFs), and six and seven putative ORFs [27]. LIR contains the origin of viral genome replication, which is essential for replication, and transcriptional elements in both the virion- and complementary-sense directions, which confer bidirectional promoter activity [30,31]. V1, C1, and C2 are the putative CP, geminivirus-like RepA, and/or Rep proteins, respectively [11] (Figure 1). Not only is V3 a putative MP but also the experimentally identified suppressor of posttranscriptional gene silencing (PTGS) [32] and pathogenicity determinant [33]. V4 enhances viral genome replication to some extent during MCLV infection, and V5 is necessary for the infection of MCLV infectious clones into *N. benthamiana* [20].

V2 is expressed from the first virion-sense ORF, which is transcribed by the promoter within the LIR sequence located upstream of the ORF. However, according to BLASTp and CD searches against the GenBank database via the amino acid sequence of V2, no analog or conserved domains were identified. The role of V2 during the MCLV infection cycle remains unknown. Here, we experimentally determined that V2 functions to increase MCLV genome DNA replication during the viral life cycle via *Agrobacterium*-mediated infectious clones of the wild-type MVLV vII constructed previously and two V2-mutated MVLV vII constructed in this study. These findings provide new insights into the functions of MCLV-encoded proteins.

## 2. Results

### 2.1. MCLV-Encoded V2 Enhances MCLV Genome Replication in Plants

**Figure 2:**
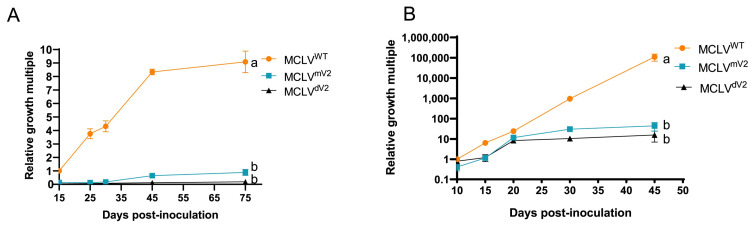
Real-time quantitative PCR (qPCR) analysis of MCLV accumulation in the systemic leaves of mulberry (**A**) and tomato (**B**) plants inoculated with pCA-1.1MCLV^WT^, pCA-MCLV^mV2^, or pCA-MCLV^dV2^. Data are the means of three independent biological replicates. Error bars represent the standard deviation (SD). The samples with the same letter are not significantly different (*p* < 0.05), according to the least significant difference’s (LSD’s) multiple comparison test. Different letters represent significant differences at *p* < 0.05 probability level.

To investigate the roles of MCLV-encoded V2 during MCLV infection, *Agrobacterium*-mediated infectious clones of wild-type MCLV^WT^ (pCA-1.1MCLV^WT^) and V2-mutated MCLV^mV2^ and MCLV^dV2^ (pCA-MCLV^mV2^ and pCA-MCLV^dV2^) were constructed and inoculated into mulberry and tomato plants, respectively. The copy number of the MCLV *CP* gene was quantitatively analyzed via qPCR, and the relative growth multiples were used to evaluate the accumulation level of the virus in the plants. The relative growth multiples of MCLV^mV2^, MCLV^dV2^, and MCLV^WT^ in either inoculated mulberry or tomato plants increased over days postinoculation (dpi), but the magnitude of the increase varied significantly (Figure 2). The accumulation levels of progeny MCLV^mV2^ and MCLV^dV2^ in both tomato and mulberry plants inoculated with pCA-MCLV^mV2^ and pCA-MCLV^dV2^ were maintained at a low level throughout the survey and were consistently significantly lower than those of the progeny MCLV^WT^ in those inoculated with pCA-1.1 MCLV^WT^. MCLV^mV2^ accumulated slightly more than MCLV^dV2^ did in both mulberry and tomato plants. More specifically, the relative growth multiples of progeny MCLV^mV2^ and MCLV^dV2^ in mulberry plants were approx. 0.9- and 0.2-fold, respectively, which were significantly lower than nine-fold of progeny MCLV^WT^ in mulberry plants at 75 dpi (Figure 2A). The relative growth multiples of progeny MCLV^mV2^ and MCLV^dV2^ in tomato plants were approx. 44- and 16-fold, respectively, which were significantly lower than 110,000-fold of progeny MCLV^WT^ in tomato plants at 45 dpi (Figure 2B). In contrast, although the relative growth multiples of progeny MCLV^mV2^ in both mulberry and tomato plants was consistently greater than that of progeny MCLV^dV2^ in both plants inoculated with pCA-MCLV^dV2^, there was no significant difference between them (Figure 2A,B).

The genome sequencing analysis of MCLV in the systemic leaves of the inoculated mulberry plants at 75 dpi and tomato plants at 45 dpi confirmed that the progeny MCLV^mV2^ was derived from the pCA-MCLV^mV2^ construct, MCLV^dV2^ from pCA-MCLV^dV2^, and MCLV^WT^ from pCA-1.1MCLV^WT^. Symptom observation for the inoculated mulberry plants at 75 dpi and the inoculated tomato plants at 45 dpi showed that neither MCLV^WT^-positive nor MCLV^mV2^- and MCLV^dV2^-positive plants expressed obvious virus-like symptoms on the leaves.

These results indicated that the absence of V2 may significantly downregulate the replication of MCLV genomic DNA during MCLV infection in plants. V2 is not obviously associated with the systemic infection of MCLV and symptom expression of MCLV-infected hosts.

### 2.2. MCLV-Encoded V2 Enhances MCLV Genome Replication at the Cellular Level

**Figure 3:**
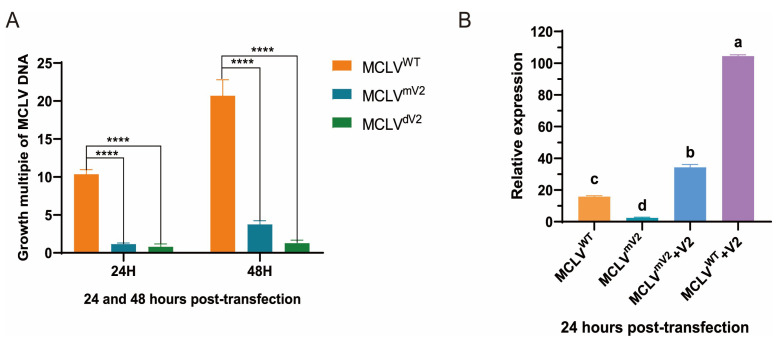
qPCR analysis of MCLV replication in protoplasts transfected with pCA-1.1MCLV^WT^, pCA-MCLV^mV2^, and pCA-MCLV^dV2^ (**A**) and with pCA-1.1MCLV^WT^, pCA-MCLV^mV2^, pCA-MCLV^mV2^ + pRI-V2, and pCA-1.1MCLV^WT^ + pRI-V2 (**B**). The growth multiple was calculated by the formula of the copy number at 24 h/0 h and 48 h/0 h post-transfection in different samples. The data are the means of three independent biological replicates. Error bars represent the standard deviation (SD). Asterisks indicate a statistically significant difference according to the unpaired Student’s *t*-test (two-tailed); **** *p* < 0.0001. Analysis of significant difference (*p* < 0.05) was carried out using the LSD’s multiple comparison test. Different letters in subfigure B represent significant differences at *p* < 0.05 probability level.

To investigate whether V2 enhances the replication of the MCLV DNA genome at the cellular level, three constructed recombinant plasmids, pCA-1.1MCLV^WT^, pCA-MCLV^mV2^, and pCA-MCLV^dV2^, were transfected into protoplasts of *N. benthamiana*, respectively (Figure S2). The copy number of the MCLV vII *CP* gene in protoplasts at 0, 24, and 48 h post-transfection (hpt) was quantified via qPCR, and the growth multiples were used to assess the accumulation levels of each MCLV. The growth multiples of MCLV^mV2^ in protoplasts transfected with pCA-MCLV^mV2^ were approx. 1.2- at 24 hpt and 3.8-fold at 48 hpt, and those of MCLV^dV2^ were 0.7- at 24 hpt and 1.3-fold at 48 hpt, respectively, which were significantly lower than 10-fold of MCLV^WT^ at 24 hpt and 21-fold at 48 hpt (Figure 3A). These results indicated that V2 plays a positive role in increasing MCLV genome replication at the cellular level, as MCLV accumulation was strongly reduced in protoplasts with V2-deficient MCLV.

### 2.3. Expression of V2 Is Strongly Related to Replication of the MCLV DNA Genome

To further determine whether the absence of V2 is responsible for the downregulation of replication of the V2-mutant MCLV genome, a complement experiment was carried out by co-transfecting protoplasts with pCA-MCLV^mV2^ and the V2 expression plasmid pRI-V2. qPCR revealed that the accumulation of the virial genome was approximately 8-fold lower in the protoplasts transfected with pCA-MCLV^mV2^ but 2.3-fold greater in those co-transfected with pCA-MCLV^mV2^ + pRI-V2 than in those transfected with pCA-1.1MCLV^WT^ at 24 h post-infiltration (hpi). Sequencing analysis of the V2 ORF region of the progeny MCLV^mV2^ in protoplasts co-transfected with pCA-MCLV^mV2^ + pRI-V2 at 24 dpi confirmed that the mutated V2 remained in the genome of the progeny MCLV^mV2^. These results indicated that the downregulation of replication of the V2-mutant MCLV genome in cells was caused by the absence of V2.

**Figure 4:**
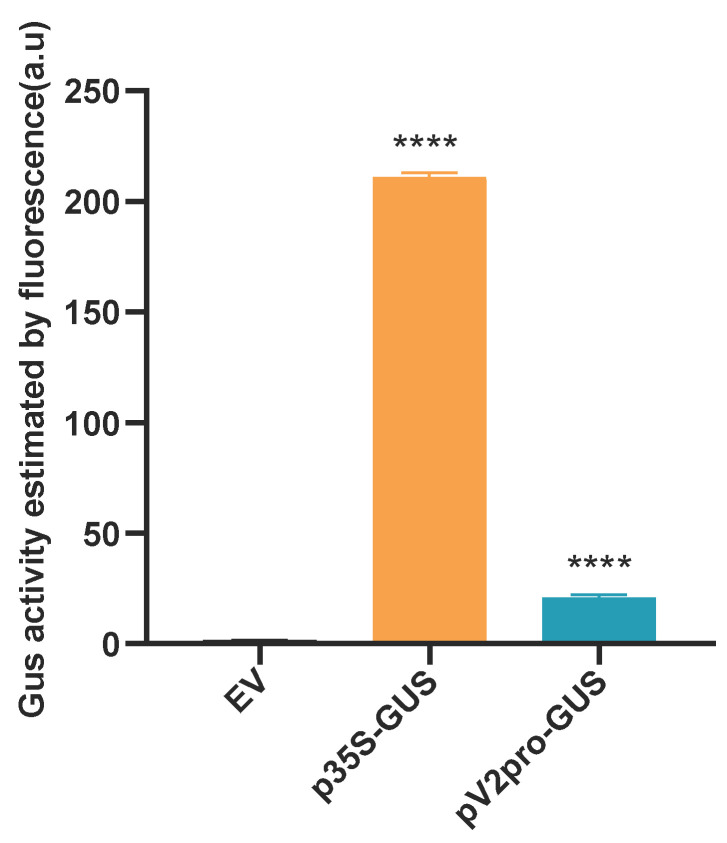
Quantitative analysis of V2 ORF promoter activity. The promoter activity was estimated by the measured fluorescence value of GUS in the different samples. EV: empty vector. The data are the means of three independent biological replicates. The bars represent the means ± standard deviations (SDs). Asterisks indicate a statistically significant difference according to the unpaired Student’s *t*-test (two-tailed); **** *p* < 0.0001.

However, an interesting phenomenon was observed in the complementation experiment, where MCLV accumulation in the protoplasts co-transfected with pCA-MCLV^mV2^ + pRI-V2 was c. 2.3 times greater than that of pCA-1.1MCLV^WT^-transfected cells at 24 dpi and, in the case of pCA-1.1MCLV^WT^ +pRI-V2, even c. 7 times higher (Figure 3B). This may be attributed to the differential expression of V2 in the respective expression systems, and this differential expression is reflected in the differences in the respective promoter activities. To verify our speculations, we compared the activity of the promoter (V2pro) driving V2 expression in the MCLV genome and the cauliflower mosaic virus (CaMV) 35S promoter (35S) driving V2 expression in the plant binary expression vector pRI 101-AN via the constructed pV2pro-GUS and p35S-GUS. The activity of V2pro was approximately 10 times lower than that of 35S (Figure 4), indicating that the expression level of V2 in the MCLV genome system was lower than that in the transient expression system. Thus, we confirmed that the greater expression of V2 in the protoplasts co-transfected with pCA-MCLV^mV2^ + pRI-V2 resulted in greater replication enhancement than in those transfected with pCA-1.1MCLV^WT^, supporting our hypothesis. These results also indicated that V2 positively regulates the replication of the MCLV genome.

## 3. Discussion

*Mulcrilevirus* is a genus (the family *Geminiviridae*) recently established with mulberry crinkle leaf virus (MCLV) as the representative species [2] and currently includes two virial species, MCLV [11] and paper mulberry leaf curl virus 1 (PMLCV-1) [12]. Interestingly, both MCLV and PMLCV-1 were identified in plants from different genera of the family *Moraceae*, MCLV from the genus *Morus*, and PMLCV-1 from the genus *Broussonetia.* The genome of mulcrileviruses encode six to seven canonical proteins on both the virion-sense strands and the complementary-sense strands [11,20,27]. Except for V2, the functions of other MCLV-encoded proteins have been determined via derivation methodologies and experiments. On the basis of the results of this study, (i) the absence of V2 significantly downregulates the replication of the MCLV DNA genome in both plants and protoplasts, (ii) complementation experiments revealed that the absence of V2 is responsible for the downregulation of replication of the V2-mutant MCLV genome, and (iii) the expression of V2 strongly affects the increase in replication of the MCLV DNA genome, and we concluded that V2 enhances the replication of the MCLV DNA genome during MCLV infection. Geminivirus-encoded proteins are generally multifunctional to compensate for their limited encoding capacity because of the small size of their genome [17,34]. Here, we revealed only one of the functions of V2, and whether other functions exist remains to be further studied.

Like V2, V4 has been previously reported to also function to increase MCLV DNA genome replication [20]. However, the V2 enhancement is twice as strong as that of V4. This feature of MCLV-encoded V2 strongly enhancing viral genome replication during viral infection is similar to that reported for begomovirus- and curtovirus-encoded C3/AC3, where the C3 protein enhances viral replication and gene expression in *N. benthamiana* and is a replication enhancer (REn) protein required for the efficient proliferation of viral DNA [15,19,35]. Therefore, we designate V2 as the REn of MCLV. Although MCLV-encoded V2 and V4 and begomovirus- and curtovirus-encoded C3 assume the function of enhancing viral genome DNA replication, there are differences in multiple aspects. A series of analyses for V2 and V4, as well as C3, revealed neither the presence of shared conserved motifs nor significant amino acid sequence similarity. Additionally, V2 and V4 are encoded by ORFs in the virion-sense strand of MCLV, whereas C3 is in the complementary-sense strand of the members of the genera *Begomovirus* and *Curtovirus*.

Geminiviruses are replicated in the nucleus of infected plant cells through rolling-circle and recombination-dependent replication (RDR) models via the plant host DNA replication machinery [36,37,38,39,40,41,42]. To optimize replication, most geminiviruses encode a REn protein [18]. The begomovirus-encoded C3/AC3 is the first identified REn protein [19]. Comparative analysis of the genome organization of geminiviruses in the 14 genera established currently [1] revealed that the geminiviruses in six genera (the genera *Begomovirus*, *Curtovirus*, *Maldovirus*, *Opunvirus*, *Topocuvirus*, and *Turncurtovirus*) contain C3 or positional homologs of C3. C3 enhances genome DNA accumulation by interacting with C1, itself, and host-encoded proteins, such as proliferating cell nuclear antigen (PCNA) [18] and the subunit of the nuclear replicative DNA polymerase δ [41], in geminivirus replication. The MCLV genome does not encode a positional homolog of the begomovirus-encoded C3; instead, V2 performs the function of REn. Searches of the ORF against the genome of PMLCV-1 (GenBank accession No. NC_076464) [12], another virus species in the genus *Mulcrilevirus*, also did not reveal the presence of an ORF for the C3 positional homolog. V2 presents no significant amino acid sequence similarity to other proteins deposited in the GenBank database. It is impossible to infer how V2 enhances MCLV genome DNA replication on the basis of its similarity to proteins of known activity. Thus, the mechanism by which V2 enhances MCLV genome DNA replication requires further elucidation.

Mutations in the MCLV V2 ORF do not prevent infection of pCA-MCLV^mV2^ and pCA-MCLV^dV2^ to natural host mulberry plants and experimental host tomato plants, suggesting that V2 does not affect the systemic movement of the progeny MCLV in infected plants. The begomovirus-encoded REn protein greatly enhances not only viral DNA genome accumulation but also disease symptoms in geminivirus-infected plants [43,44]. However, observations of symptoms in mulberry plants at 100 dpi and tomatoes at 90 dpi indicate that, like MCLV^WT^ [28,29], both MCLV^mV2^ and MCLV^dV2^ also do not induce any virus-like symptoms on the systemic leaves of infected plants. These findings suggest that there is no obvious correlation between V2 and systemic movement, infectivity, or symptom induction of MCLV.

The geminivirus genes can be divided into early and late genes according to their timing of expression during viral infection [16,25]. The early genes include those that drive replication and transcription of the geminiviral DNA genome, and the late genes are involved in encapsidation and movement of geminiviruses [45]. For example, geminivirus-encoded Rep, C2, and C3 (REn) are early genes [46,47], whereas CP is a late gene [46]. This finding suggested that the genes encoded by the geminivirial complementary-sense strand are early genes, whereas those encoded by the virion-sense strand are late genes [16]. However, a recent report is not consistent with this idea, as V3, which is encoded by the virion-sense strand of TYLCV, is an early gene [25]. In this study, V2 is encoded by the virion-sense strand of MCLV, and its expression is driven by the promoter located in the LIR, which has bidirectional promoter activity. The promoter of V2 was active in the absence of infection, suggesting that it is also an early gene, despite its expression in the virion-sense strand of MCLV.

Both mutation and deletion of the *V2* gene resulted in a decrease in the genome replication of MCLV, but the reduction caused by V2 deletion was greater than that by V2 mutation. The greatest possibility responsible for this result is that the deleted sequence in the V2 deletion mutant causes V3 expression to be affected to some extent, thereby affecting genome replication indirectly by an unknown mechanism during MCLV infection.

In conclusion, V2 is a REn protein of MCLV. Its expression strongly enhances the replication of the MCLV DNA genome during its life cycle. However, the deletion of V2 does not alter the systemic infection or pathogenicity of MCLV during infection. These findings are helpful not only for further research on the biology but also for the utilization of MCLV, such as the development of a virus-induced gene silencing (VIGS) vector based on MCLV.

## 4. Materials and Methods

### 4.1. Preparation of Plant Materials

Healthy mulberry seedlings used in this study were obtained from seeds collected from a mulberry tree (cultivar Yu711) with neither virus-like symptoms nor any plant viruses confirmed by high-throughput sequencing (HTS). The mulberry seedlings, along with *Nicotiana benthamiana* and *Solanum lycopersicum* (tomato) plants, were cultivated in a temperature- and light-controlled room with a constant temperature of 26 °C, 50–60% relative humidity, and a 16-/8-h light/dark cycle.

### 4.2. Construction of Agrobacterium-Mediated Infectious Clones of V2 Mutant MCLV

Two MCLV variants, MCLV [11] and MCLV vII [27], were identified in naturally infected mulberries. Since *Agrobacterium*-mediated infectious clones of MCLV vII infect not only mulberry and tomato but also *N. benthamiana*, while that of MCLV infects mulberry and tomato but not *N. benthamiana*, MCLV vII was used as a wild-type MCLV (MCLV^WT^) in this study. An *Agrobacterium*-mediated infectious clone of MCLV vII (pCA-1.1MCLV^WT^) previously constructed by our laboratory [29] and the promoter-free vector pCAMBIA2300 were employed to construct *Agrobacterium*-mediated infectious clones of two V2 mutant MCLV vII (MCLV^mV2^ and MCLV^dV2^). In the V2 ORF, there are two start codons (ATG) that can form two ORFs, which form an ORF-encoding V2 based on the first ATG and forms another ORF-encoding N-terminal truncated V2 based on the second ATG. For the MCLV^mV2^ construct, these two ATG were mutated to GTG together (Figure S1). For the MCLV^dV2^ construct, only 133 nucleotides at the 5′-end of the V2 ORF were deleted, because the remaining 185 nucleotides (nt 134–318) overlap completely with the V3 ORF (Figure S1). The constructions of pCA-MCLV^mV2^ and pCA-MCLV^dV2^ were carried out as previously described [20]. Briefly, the nucleotide sequences of MCLV^mV2^ and MCLV^dV2^ were amplified from pCA-1.1MCLV^WT^ using high-fidelity polymerase, PrimeSTAR^®^ GXL DNA Polymerase (TaKaRa, Beijing, China), and the primers MCLV-tF1/MCLV-mV2R1 and MCLV-mV2F2/MCLV-tR2; MCLV-tF1/MCLV-dV2R1 and MCLV-dV2F2/MCLV-tR2 (Table S1), respectively. The target fragments were inserted into *Sal* I-digested pCAMBIA2300 via seamless cloning using the ClonExpress MultiS One Step cloning kit (Vazyme, Nanjing, China). The resulting constructs were then transformed into *Escherichia coli* DH5α competent cells and verified by sequencing (SUNYA, Hangzhou, China). All primers used in this study were synthesized by GenScript Biotech Co., Ltd. (Nanjing, China).

### 4.3. Construction of the Transient Expression Recombinant Plasmid

To construct a recombinant plasmid for V2 transient expression, the full-length nucleotide sequence (318 nt) of the V2 ORF was PCR amplified from pCA-1.1MCLV^WT^ using PrimeSTAR^®^ GXL DNA Polymerase (TaKaRa) and the primers PRI-V2F/PRI-V2R (Table S1). PCR products were separated via 1% agarose gel electrophoresis. The target fragment was reclaimed using a SanPrep Column DNA Gel Extraction Kit (Sangon Biotech, Shanghai, China) and inserted into *Sal* I-digested pRI 101-AN (TaKaRa) using the ClonExpress MultiS One Step cloning kit (Vazyme). The resulting construct was transformed into *E. coli* DH5α, verified by sequencing (SUNYA) and named pRI-V2.

To assess the promoter activity of the *V2* gene, the upstream sequence (LIR of MCLV) of the V2 ORF was PCR-amplified from pCA-1.1MCLV^WT^ using the primers pF/pR, with *Hind* III and *Bam* HI sites (Table S1), pF CCGAAGCTTTTATAGAAGGGAAGGAGTTG (the underlined sequence is the *Hind* III site), and pR ATTGGATCCCGGTTCCTTGCTCCGC (the underlined sequence is the *Bam* HI site). The PCR products were separated via 1% agarose gel electrophoresis. The target fragment (346 bp) was reclaimed using the SanPrep Column DNA Gel Extraction Kit (Sangon), inserted into the pGEM-T easy vector (Promega Corp., Madison, WI, USA), and verified by sequencing (SUNYA). The correct inserts were double-digested with *Hind* III and *Bam* HI and inserted into the same sites of the promoter–capture binary vector pCAMBIA1391z upstream of the β-glucuronidase (GUS) reporter gene, producing a pCAMBIA1391z-V2pro-GUS (pV2pro-GUS) construct. pCAMBIA1391z-CaMV 35S (p35S-GUS), constructed previously in our laboratory [48], was used as a positive control.

### 4.4. Preparation and Inoculation of Agroinocula

Recombinant plasmids, including pCA-MCLV^mV2^, pCA-MCLV^dV2^, pCA-1.1MCLV^WT^, pV2pro-GUS, and p35S-GUS, were extracted from *E. coli* DH5α and transformed into *Agrobacterium tumefaciens* EHA105-competent cells by electroporation via the Gene Pulser Xcell System (Bio-Rad, Hercules, CA, USA). The transformed *Agrobacterium* cells carrying the recombinant plasmids were cultured in Luria–Bertani (LB) media supplemented with 50 μg/mL kanamycin and 50 μg/mL rifampicin in a shaker at 200 rpm and 28 °C. The cultured *Agrobacterium* cells were collected by centrifugation for 2 min at 6000 rpm and resuspended in buffer containing 0.5% (*w*/*v*) d-glucose, 50 mM 2-(N-morpholino)-ethanesulfonic acid (MES, pH 5.6), 2 mM Na_2_HPO_4_, and 100 μM acetosyringone to OD_600_ = 1.0. The resulting *Agrobacterium* cells were incubated for 3–5 h at room temperature before inoculation.

*Agrobacterium*-mediated infectious clones (pCA-1.1MCLV^WT^, pCA-MCLV^mV2^, and pCA-MCLV^dV2^) and a negative control (empty pCAMBIA2300 vector) were inoculated into mulberry and tomato seedlings via the modified “young leaf-stab inoculation” and “young stem-slash inoculation” methods, respectively [28]. The absence of MCLV in inoculated healthy mulberry seedlings was confirmed via PCR using the MCLV-specific primers MCLV-jcF/MCLV-cjR (Table S1) before inoculation.

### 4.5. Isolation and Transfection of N. benthamiana Protoplasts

Isolation of *N. benthamiana* protoplasts was performed as previously described [20]. The transfection of plasmid DNA into isolated protoplasts was conducted following the protocol established by Dai et al. [49].

### 4.6. DNA Extraction and Confirmation of the V2 ORF in Progeny MCLV

DNA was extracted from the plants and protoplasts using the Ezup Spin Column Plant Genomic DNA Extraction Kit (Sangon) following the manufacturer’s instructions. The extracted DNA from 0.1 g of plant leaf tissue was resuspended in 20 μL of double-distilled (dd) H_2_O.

To confirm the presence or absence of the V2 ORF in progeny MCLV from the samples inoculated or transfected with pCA-1.1MCLV^WT^, pCA-MCLV^mV2^, and pCA-MCLV^dV2^, PCR against the V2 ORF was performed using *Ex Taq*^®^ polymerase (TaKaRa), the primers MCLV-flaV2F/MCLV-flaV2R (Table S1) designed based on sequences flanking the V2 ORF, and DNA extracted from the respective samples. The PCR products were separated via 1% agarose gel electrophoresis in 1 × TAE buffer. The targeting fragments were purified using the SanPrep Column DNA Gel Extraction Kit (Sangon) and inserted into the pMD^TM^19-T vector (TaKaRa). The resulting plasmids were subsequently transformed into *E. coli* DH5α. The positive clones were confirmed via colony PCR using the appropriate primers, and 3 positive clones from each sample were sequenced (SUNYA).

### 4.7. Real-Time Quantitative PCR (qPCR)

DNA extracted from samples collected from the same leaves of MCLV-positive plants and from protoplasts at different time points was used as a template for absolute qPCR using the NovoStart^®^ SYBR qPCR SuperMix Plus Kit (Novoprotein, Suzhou, China). The absolute qPCR and accumulation analyses of the MCLV genome in the samples were performed as previously described [20,35]. The standard curves were established by Han [20]. All the qPCR experiments were conducted with at least three biological replicates and three technical replicates for each biological sample. The growth multiple in the protoplast essays was calculated according to the number of MCLV vII *CP* copies in different samples at the target time points divided by those at the initial time points. The relative growth multiple of the progeny MCLV was calculated by the formular of the MCLV CP copy number quantified in different sample plants at different stages after inoculation divided by that in the mulberry plants inoculated with pCA-1.1MCLV^WT^ at 15 dpi or tomato plants at 10 dpi.

### 4.8. GUS Fluorometric Assays

Agroinocula of p35S-GUS (positive control), pV2pro-GUS, and the empty vector pCAMBIA1391Z (negative control) were individually infiltrated into *N. benthamiana* leaves as previously described [33]. The infiltrated leaf patches were collected 72 h postinfiltration. A qualitative analysis of GUS activity in plant leaf tissues was carried out as previously described [48]. The promoter activity was estimated by the strength of GUS activity. Three biological replicates were performed for the assay.

## Data Availability

Data related to the findings presented in this paper are available from the corresponding authors upon reasonable request.

## Supplementary Materials

The following supporting information can be downloaded at https://www.mdpi.com/article/10.3390/ijms251910521/s1.
