# Supplementary material for: V2 Protein Enhances the Replication of Genomic DNA of Mulberry Crinkle Leaf Virus
Source: Int J Mol Sci. 2024 Sep 29;25(19):10521. doi: 10.3390/ijms251910521 (PMC11476850; doi:10.3390/ijms251910521)
Supplement: Supplementary file 1 [file ijms-25-10521-s001.zip › ijms-3144015-supplementary.pdf]

Type of the Paper (Article)

V2 Protein Enhances the Replication of Genomic DNA of Mulberry Crinkle Leaf Virus

Zhen-Ni Yin [1,+], Pei-Yu Han [1,+],Tao-Tao Han [1], Ying Huang [1], Jing-Jing Yang[1], Meng-Si Zhang [1], Miao Fang [1,2], Kui Zhong [1,2], Jian Zhang [1,2,*], and Quan-You Lu [1,2,*]

Supplemental Table S1

Supplemental Figures S1 and S2 and legends

Table S1 Primers used in this paper

Primer	Primer sequence（5'→3'）
**For construction of V2-mutant MCLV**	
MCLV-tF1	cggggatcctctagagtcgacTTGTAGAAGGGAAGGAGTTGGAA
MCLV-mV2R1	AAAATATTTGGGGCAGGGGGGTATTTATAGCTCA**C**CG
MCLV-mV2F2	CCCCCTGCCCCAAATATTTTTAAA**G**TGTC
MCLV-tR2	agggcatgcctgcaggtcgacCGGTTTCTTGCTCCGCAA
MCLV-dV2R1	AGACATCGGTTTCTTGCTCCGCAA
MCLV-dV2F2	GGAGCAAGAAACCGATGTCTTTGTGGAGTACCAAATTAGG
**For pRI-V2 construction used in complementation experiment**	
pRI-V2F	ttgatacatatgcccgtcgacATGAGCTATAAATACCCCCCTGC
pRI-V2R	tccggtacccccggggtcgacCTACGGCACTGAGTAAGGTGGAC
**For pV2pro-GUS construction**	
pF	CCGAAGCTTTTATAGAAGGGAAGGAGTTG
pR	ATTGGATCCCGGTTCCTTGCTCCGC
**For PCR detection**	
MCLV-jcF	CAGTGAAACGTGGAACATTTGGAAT
MCLV-jcR	CAATTAGCAGTCAACGTCACATTCT
**For qPCR assay**	
qMCLVF1	GCAAGTGTCATGCGAACCCC
qMCLVR1	TACCCATTACCACCAGTATG
**For amplification of full-length sequence of V2 ORF**	
MCLV-flaV2F	GCTTGCGGAGCAAGAAACCG
MCLV-flaV2R	GTCTTATCAGTCTCAACAGT

Note: The sequences with horizontal underline are the homologous arm sequence for homologous recombination between target gene and vector. The sequences with dotted underline are the homologous arm sequence for homologous recombination between target gene fragments. The red bold letters are the points of the mutation. The sequences with wavy lines are the restriction enzyme sites.

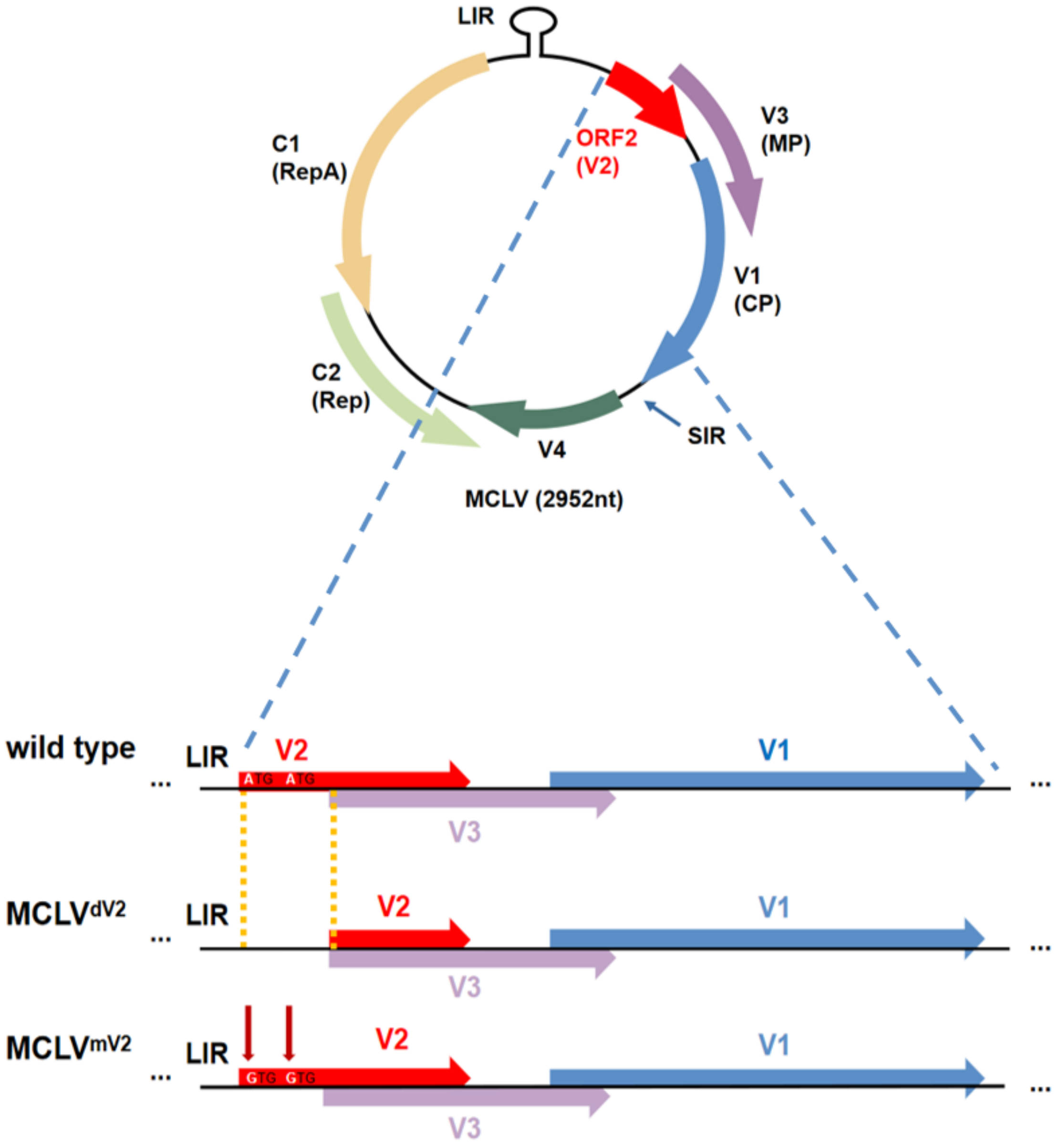

Figure S1 Schematic diagram of MCLV^{dV2} and MCLV^{mV2}. For MCLV^{dV2}, only 133 nucleotides at 5'-end of the V2 ORF were deleted because the remaining 185 nts (nt 134-318) overlap completely with V3 ORF. For MCLV^{mV2}, two start codons (ATG) within the V2 ORF were mutated to GTG.

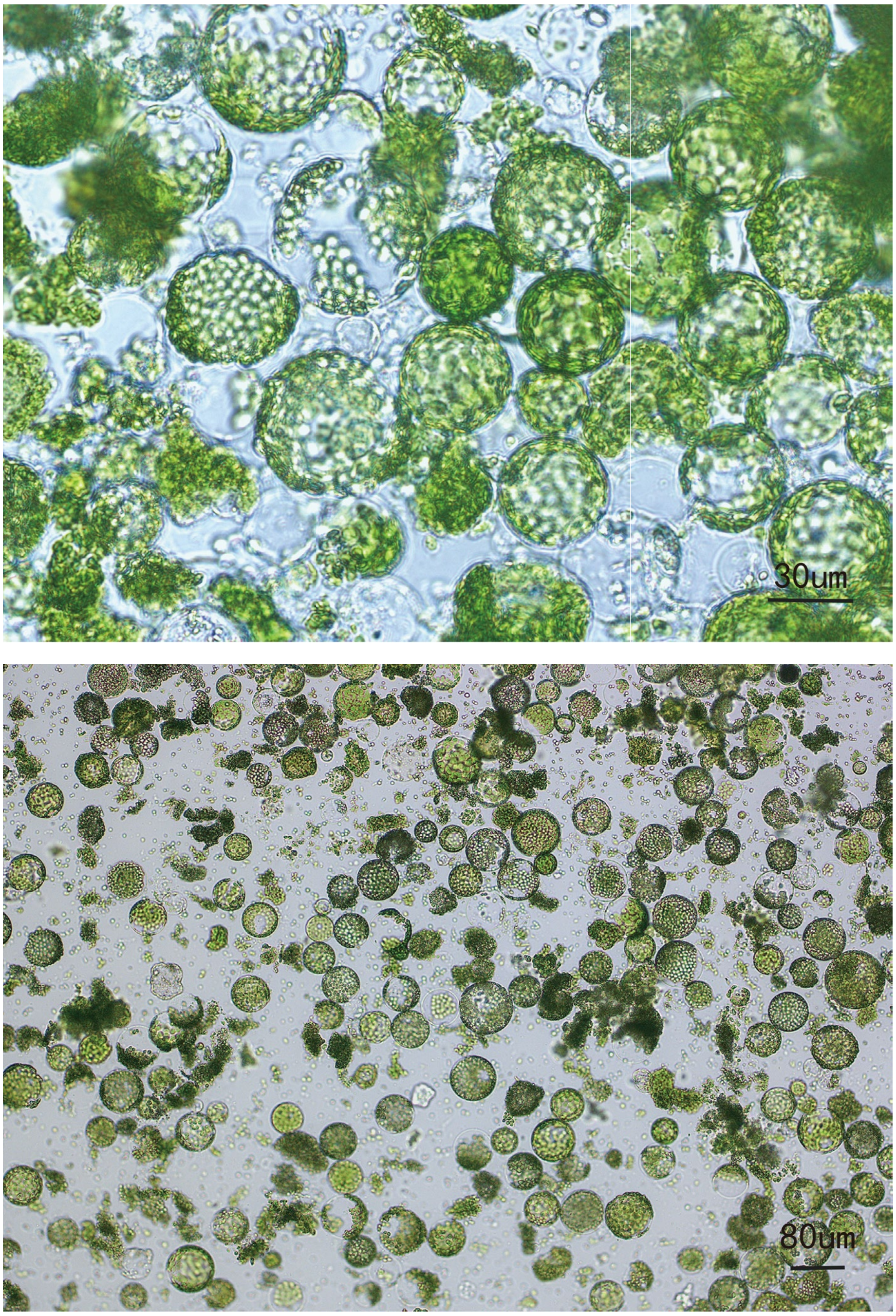

Figure S2 Images of protoplasts isolated from *N. benthamiana*
The scale bar in the upper image is 30 μm, and the lower image 80 μm.
